# Vocal correlates of sender-identity and arousal in the isolation calls of domestic kitten (*Felis silvestris catus*)

**DOI:** 10.1186/1742-9994-9-36

**Published:** 2012-12-21

**Authors:** Marina Scheumann, Anna-Elisa Roser, Wiebke Konerding, Eva Bleich, Hans-Jürgen Hedrich, Elke Zimmermann

**Affiliations:** 1 Institute of Zoology, University of Veterinary Medicine Hannover, Bünteweg 17, Hannover D-30559, Germany; 2 Institute for Laboratory Animal Science, Hannover Medical School, Carl-Neuberg-Straße 1, Hannover D-30626, Germany

**Keywords:** Affect-intensity, Individual signature, Infant, Mammal, Cat, Vocalisation

## Abstract

**Introduction:**

Human speech does not only communicate linguistic information but also paralinguistic features, e.g. information about the identity and the arousal state of the sender. Comparable morphological and physiological constraints on vocal production in mammals suggest the existence of commonalities encoding sender-identity and the arousal state of a sender across mammals. To explore this hypothesis and to investigate whether specific acoustic parameters encode for sender-identity while others encode for arousal, we studied infants of the domestic cat (*Felis silvestris catus*). Kittens are an excellent model for analysing vocal correlates of sender-identity and arousal. They strongly depend on the care of their mother. Thus, the acoustical conveyance of sender-identity and arousal may be important for their survival.

**Results:**

We recorded calls of 18 kittens in an experimentally-induced separation paradigm, where kittens were spatially separated from their mother and siblings. In the Low arousal condition, infants were just separated without any manipulation. In the High arousal condition infants were handled by the experimenter. Multi-parametric sound analyses revealed that kitten isolation calls are individually distinct and differ between the Low and High arousal conditions. Our results suggested that source- and filter-related parameters are important for encoding sender-identity, whereas time-, source- and tonality-related parameters are important for encoding arousal.

**Conclusion:**

Comparable findings in other mammalian lineages provide evidence for commonalities in non-verbal cues encoding sender-identity and arousal across mammals comparable to paralinguistic cues in humans. This favours the establishment of general concepts for voice recognition and emotions in humans and animals.

## Introduction

Human speech and non-linguistic vocalisations convey paralinguistic cues encoding the physical characteristics of a speaker, termed here indexical cues (e.g., sex, age, body size, sender-identity), and the emotional state of a sender, termed here prosodic cues (e.g., emotional valence, arousal) (e.g., [1-3]). Whereas linguistic aspects of human speech are unique to humans, non-verbal cues comparable to paralinguistic cues were also found in the vocalisations of animals of at least 11 mammalian orders (for indexical cues e.g., humans: [3,4], non-human primates: [5,6], Scandentia: [7]; Artiodactyla: [8,9]; Perissodactyla: [10,11]; Carnivora: [12,13]; Cetaceae: [14]; Chiroptera: [15,16]; Rodentia: [17,18]; Proboscidae: [19,20]; Sirenia: [21]; Hyracoidea: [22]; for prosodic cues see review [23-25]). This suggests a pre-human origin of paralinguistic cues due to homologies in the central nervous system and the mammalian vocal production system.

In mammals vocal production is based on a highly evolutionarily conserved system. According to the Source-Filter theory of vocal production the respiratory airstream from the lungs passes the larynx (=source) with the vocal folds followed by the supra-laryngeal vocal tract (=filter; [2,26,27]). Indexical cues are suggested to be related to the length, the density and the tension of the vocal folds (affecting the fundamental frequency [28] of the sound signal) and to the length and the shape of the supra-laryngeal vocal tract (affecting the formant pattern [29]). Affect-induced physiological changes are suggested to be related to changes in respiratory airstream (affecting amplitude, tempo and fundamental frequency [28,30]), changes in muscle tonus of laryngeal muscles controlling the tension of the vocal folds (causing disruption and changes of fundamental frequency [28,30,31]) and changes in the shape, the length and the filter-properties of the supra-laryngeal vocal tract (affecting formant frequencies [1,30]).

Studies in human and non-human mammals demonstrated that source- and/or filter-related acoustic parameters are important acoustic parameters encoding sender-identity (e.g. [4,6]), whereas time-, source- and tonality-related variations are associated with the arousal of the sender (e.g., [23,24,32-34]). Furthermore, non-linear phenomena (NLP), irregular vibrations of the vocal folds (e.g., subharmonics, biphonations, frequency jumps), have become a focal point of acoustic research describing highly complex vocalisations (e.g., [35-39]) and are common in human and non-human animals [35-37,39-42]. However, their function is not yet clear [36,39,43]. On the one hand, it is argued that NLPs could be important for individual recognition (e.g., [36,37,39,42]) and on the other hand that NLPs convey information about the emotional state of the sender (e.g. [37,39]).

To explore the impact of certain acoustic parameters on encoding sender-identity and arousal in non-human mammals, it is important to study both aspects in the same individuals using the same set of acoustic parameters and the same behavioural contexts. To date, there are only few studies investigating both aspects in the same individuals and behavioural contexts (bats: [44,45]; primates: [30,46]; elephants: [20]; dogs: [47]; tree shrews: [7]) and to our knowledge only three studies are available for mammalian infants (elephants: [37]; bats: [45]; cattle: [48]). To explore the role and potential commonalities of certain acoustic parameters or sets of acoustic parameters encoding prosodic and indexical cues in mammalian infant vocalisations, further studies on infants of various mammalian taxa are needed.

In this study, we explored vocal cues encoding sender-identity (indexical cues) and arousal (prosodic cues) by investigating infant isolation calls of domestic cats. Cats are an important animal model in human hearing research due to similarities in their auditory system to humans (e.g. [49,50]). Adult females usually live communally in small social groups, whereas males live solitarily [51]. Domestic cats are an altricial species, kittens being born blind with their ears closed [52]. During the first three weeks after birth visual and auditory skills of the kittens as well as their locomotor and thermoregulatory abilities are limited [52-54] and kittens are completely dependent on their mother. Cats have an elaborated vocal repertoire [55-59]. Thus, infant vocalisations may play an important role for their survival, signalling their emotional state and their needs. Females give birth to one to 10 infants per litter [51]. Litters from different females may be reared in the same nest and thus, may become mixed, which could make kin signatures essential for offspring recognition and offspring-directed maternal care [51]. Previous studies have already shown that kittens produce isolation calls when isolated from their mother [55,57-60] which evoke maternal behaviour [61]. Context and age-specific variations in the acoustic structure of kitten isolation calls have already been described but only for a few acoustic parameters [58,60], whereas to our knowledge no data on acoustically conveyed individual signatures in kitten isolation calls have been published.

The aim of this study was to investigate the following two hypotheses: (1) sender-identity is encoded in the acoustic structure of kitten isolation calls, (2) arousal is encoded in the acoustic structure of kitten isolation calls and non-linear phenomena occur more often in High arousal compared to Low arousal situations. Based on these results we aimed to investigate which acoustic parameters or sets of acoustic parameters are important for encoding sender identity and which are important for encoding arousal. Vocal correlates of arousal in non-human animals can be investigated at the behavioural level by measuring different levels of situational urgency within the same behavioural context and linking it to the corresponding vocal expression [23]. Thus, we separated the kittens from their mother and siblings and exposed them to two sub-contexts which were assumed to vary in their level of arousal (Low arousal versus High arousal condition). To investigate our hypotheses multi-parametric sound analyses were performed measuring 3 time-, 4 source-, 12 filter- and 3 tonality-related parameters (Table 1). We will report that a set of source- and filter-related acoustic parameters is important for encoding sender-identity, whereas a set of time-, source- and tonality-related acoustic parameters is important for encoding arousal. By comparing our findings with data on other mammals we will explore to which extent our results support the hypothesis for universal acoustic coding rules expressing indexical and prosodic cues in mammals due to similar physiological and anatomical constraints in the peripheral vocal production system.

**Table 1:** Description of measured acoustic parameters

**Parameter**	**Definition**
**Time-related parameters**	
Call duration [ms]	Time between the onset and the offset of a call.
ICI [ms]	Time between the offset of a call and the onset of the successive call.
Peaktime [ms]	Time between the onset and the maximum amplitude of a call.
**Source-related spectral parameters**	
MeanF0 [Hz]	Mean fundamental frequency of a call.
MinF0 [Hz]	Minimum fundamental frequency of a call.
MaxF0 [Hz]	Maximum fundamental frequency of a call.
SDF0 [Hz]	Standard deviation of the fundamental frequency of a call.
**Filter-related spectral features**	
Peak [Hz]	Frequency with maximum energy over a call.
MeanF1 [Hz]	Mean frequency of the first formant of a call.
SDF1 [Hz]	Standard deviation of the first formant frequency of a call.
BWF1[Hz]	Bandwidth of the first formant frequency of a call.
MeanF2 [Hz]	Mean frequency of the second formant of a call.
SDF2 [Hz]	Standard deviation of the second formant frequency of a call.
BWF2 [Hz]	Bandwidth of the second formant frequency of a call.
MeanF3 [Hz]	Mean frequency of the third formant of a call.
SDF3 [Hz]	Standard deviation of the third formant frequency of a call.
BWF3 [Hz]	Bandwidth of the third formant frequency of a call.
F2–F1 [Hz]	Difference between the mean of the second and the first formant frequency.
Consistency	Mean maximum correlation of power spectra of successive 25 ms time steps of a call.
**Tonality-related parameters**	
Cepstral peak [V]	Value of the peak at the fundamental period of a cepstrum for the middle 10 ms of the call.
Voiced [%]	Percentage of voiced frames of a call.
MaxHNR [db]	Maximum harmonic-to-noise ratio of a call.

## Results

We found no significant differences in the acoustic parameters between individuals which were initially exposed to the Low or the High arousal condition (Fishers Omnibus test: χ^2^≤55.55, df=44, p≥0.114 for both conditions). This suggests that the order in which the subjects were exposed to the two arousal conditions did not affect the acoustic parameters of their vocalizations. Therefore, both groups were pooled for further analysis.

### Sender-identity

For both arousal conditions the majority of time-, source-, filter- and tonality-related parameters showed significant differences between individuals (Fisher Omnibus test: χ^2^≥784.64, df=44, p<0.001; Table 2). For time-related parameters almost all parameters differed significantly between individuals for both arousal conditions (High arousal: F(17)≥1.89, N=18, p≤0.022; Low arousal: F(15)≥2.69, N=16, p≤0.001 except ICI F(15)=1.23, N=16, p=0.256). For the source- and tonality-related parameters all measured acoustic parameters differed between individuals for both arousal conditions (Low arousal: F(15)≥2.57, N=16, p≤0.002; High arousal: F(17)≥1.96, N=18, p≤0.016). For the filter-related parameters almost all measured parameters for both arousal conditions differed between individuals (High arousal: F(17)≥1.90, N=18, p≤0.022; Low arousal: F(15)≥1.85, N=16, p≤0.033 except BWF2 and SD3: F(15)≤1.73, N=16, p≥0.052). To investigate whether calls can correctly classified to the respective individuals, we performed Discriminant function analysis (DFA) combined with Principal Component Analysis (PCA) for each arousal condition separately.

**Table 2:** Results of the one-way Anova testing for differences between individuals for each acoustic parameter and arousal condition and the correlation coefficient with the three most important PCs for the DFA; LOW = Low arousal condition; HIGH = High arousal condition; bold p-values represent significant difference p < 0.05; bold loading factors represent the parameters showing loading factors higher than 0.700 with the respective PC

	**LOW**					**HIGH**				
**Parameters**	**F**	**p**	**PC1**	**PC2**	**PC6**	**F**	**p**	**PC1**	**PC2**	**PC3**
**Time-related parameters**										
Call duration [ms]	6.632	**<.001**	-.368	.365	.324	5.574	**<.001**	-.147	-.375	.132
ICI [ms]	1.230	0.256	-.080	-.113	-.218	1.894	**0.022**	.255	.168	.124
Peaktime [ms]	2.688	**0.001**	-.203	.056	.487	3.978	**<.001**	-.032	-.412	.145
**Source-related parameters**										
MeanF0 [Hz]	25.331	**<.001**	**.863**	.047	.304	20.199	**<.001**	-.380	**.810**	.018
MinF0 [Hz]	16.034	**<.001**	**.864**	-.149	.050	10.574	**<.001**	-.400	.650	-.312
MaxF0 [Hz]	27.394	**<.001**	**.751**	.213	.426	17.166	**<.001**	-.341	**.818**	.166
SDF0 [Hz]	2.806	**0.001**	-.361	.363	.380	5.921	**<.001**	.012	.371	.602
**Filter-related parameters**										
Peak [Hz]	3.919	**<.001**	.387	.255	.132	10.444	**<.001**	**-.725**	-.236	.071
MeanF1 [Hz]	8.305	**<.001**	.207	**.814**	-.081	8.631	**<.001**	-.677	-.202	.403
SDF1 [Hz]	3.170	**<.001**	-.122	**.755**	.048	4.558	**<.001**	-.047	.172	.614
BWF1 [Hz]	1.848	**.033**	-.015	.582	-.311	1.953	**.017**	.207	.112	.486
MeanF2 [Hz]	4.287	**<.001**	-.668	.078	.326	11.260	**<.001**	**.711**	.315	.174
SDF2 [Hz]	2.322	**.005**	-.147	.289	-.080	4.707	**<.001**	.406	-.133	.414
BWF2 [Hz]	1.130	.335	-.065	-.146	.072	1.896	**.022**	.281	-.143	.031
MeanF3 [Hz]	3.411	**<.001**	-.538	.022	.047	5.251	**<.001**	.658	.170	.046
SDF3 [Hz]	1.727	.052	-.247	.306	.102	2.442	**.002**	.107	-.451	.455
BWF3 [Hz]	2.086	**.014**	-.152	-.363	.047	2.386	**.003**	-.031	-.313	.045
F2-F1 [Hz]	6.789	**<.001**	**-.704**	-.385	.333	14.675	**<.001**	**.850**	.329	-.072
Consistency	2.072	**0.014**	.205	-.474	.195	5.135	**<.001**	.140	-.110	-.695
**Tonality-related parameters**										
Cepstral peak [V]	3.902	**<.001**	.174	.632	.038	3.501	**<.001**	-.230	.074	.492
Voiced [%]	2.569	**0.002**	.459	-.061	.090	1.963	**0.016**	.091	.351	.025
MaxHNR [db]	4.058	**<.001**	.556	-.197	.171	2.174	**0.007**	-.438	.169	-.156

For the Low arousal condition a PCA based on the acoustic parameters extracted seven factors (PC) with an eigenvalue higher than 1 explaining 71.95% of the variance (see Additional file 1). An independent DFA based on these seven PCs was able to classify 53.13% of the calls to the respective individual (cross-validation: 41.88%) which was significantly above chance level (6%; p<0.001). On an individual level for 15 out of 16 subjects for the original classification and for 12 out of 16 subjects for the cross-validation significantly more calls were correctly classified than expected by chance (p≤0.019). The DFA calculated seven DFs. Thereby, DF1, 2 and 3 explained 86.6% of the variation in the calls. DF1 showed the highest correlation to PC1 (r=0.568), DF2 showed the highest correlation to PC6 (r=0.698), whereas DF3 showed the highest correlation to PC2 (r=−0.593). PC1 showed the highest loading factors to the source-related parameters: MeanF0, MinF0 and MaxF0 (r≥0.751; Table 2) and to the filter-related parameter F2-F1 (r=−0.704). PC2 showed the highest correlation to the filter-related parameters: MeanF1 and SDF1 (r≥0.755). PC6 showed no loading factors above 0.700.

For the High arousal condition a PCA based on the acoustic parameters extracted seven factors with an eigenvalue higher than 1 explaining 68.90% of the variance (see Additional file 1). An independent DFA based on these seven PCs was able to classify 63.33% of the calls to the respective individual (cross-validation: 47.78%) which was significantly above chance level (6%; binomial test: p<0.001). On an individual level for all subjects for the original classification and for 16 out of 18 subjects for the cross-validation significantly more calls were correctly classified than expected by chance (p≤0.019). The DFA calculated seven DFs. Thereby, DF1, 2 and 3 explained 82.9% of the variation in the calls. DF1 showed the highest correlation to PC1 (r=−0.730), DF2 showed the highest correlation to PC2 (r=0.700), whereas DF3 showed the highest correlation to PC3 (r=0.706). PC1 showed the highest loading factor to the filter-related parameters: Peak, MeanF2, F2-F1 (r≥0.711; Table 2). PC2 showed the highest loading factor to source-related parameters: MeanF0 and MaxF0 (r≥0.810). PC3 showed no loading factor above 0.700 to any of the acoustic parameters.

Comparing the classification accuracy between both arousal conditions showed no significant differences (original: t(15)=1.29, N=16, p=0.215; cross-validation; t(15)=0.426, N=16, p=0.676) demonstrating that the level of individual distinctiveness was similar for both arousal conditions. Performing a crossed pDFA investigating differences between subjects by controlling for the arousal level also revealed that individuals could significantly correctly be classified (original: p=0.004; cross-validation: p=0.002).

Performing a nested pDFA testing for differences between subjects by controlling for litter confirmed significant differences between individuals (original and cross-validation: p≤0.001 for both arousal conditions). This suggests that individual differences cannot be explained by the fact that we used a varying number of kittens per litter so that one litter can contribute more to the results than another.

We found almost no significant differences in the acoustic parameters between sexes and almost no significant correlations with body weight. For the factor sex in the High arousal condition only the BWF3 and in the Low arousal condition only the SDF2 and SDF3 differed significantly between sexes (t(16)≥|2.45|, N=18, p≤0.026). For the factor body weight a significant negative correlation with call duration and a significant positive correlation for the percentage of voiced frames was found only for the Low arousal condition (r≥|0.540|, N=18, p≤0.021). However, controlling for multiple testing, using the Fisher Omnibus test, showed that these differences could be explained by chance (sex: χ^2^=104.33, df=88, p=0.113; body weight: χ^2^ =101.09, df=88, p=0.161). This indicates that individual differences cannot be explained by sex or body weight. Furthermore, the body weight of kittens did not differ between sexes (t(16)=1.09, N_female_=N_male_=9, p=0.292).

All in all, almost all measured acoustic parameters differed between individuals for both arousal conditions. However, classification of individuals was mainly attributed to source- and filter-related parameters. Thereby, the parameters which seem to be most important for classification were similar across conditions, suggesting consistency across different arousal levels.

### Arousal

**Figure 1:**
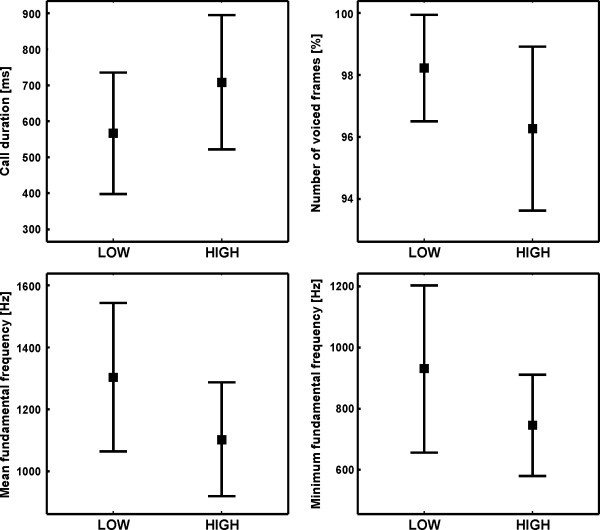
Mean and standard deviation for the Low and High arousal condition for the acoustic parameter of kitten isolation calls which had important impact on the classification of arousal; t(17)≥|2.53|, N=18, p≤0.022.

Arousal affects acoustic structure of kitten isolation calls for time-, source-, filter- and tonality-related parameters (Fisher Omnibus test: χ^2^=175.55, df=44, p<0.001; Table 3). For time-related parameters two out of three parameters differed significantly between arousal conditions. Thereby, call duration was longer in the High than in the Low arousal condition, whereas ICI was shorter (t(17)≥|2.58|, N=18, p≤0.019; Figure 1). Peaktime showed a tendency to be longer in the High than in the Low arousal condition (t(17)=−1.92, N=18, p=0.072). For the source-related parameters three out of four parameters differed significantly between conditions. Thus, the MeanF0, MinF0 and MaxF0 were lower in the High compared to the Low arousal condition (t(17)≥3.12, N=18, p≤0.006; Figure 1). For the filter-related parameters six out of 12 parameters differed significantly between conditions. Thus, Peak and MeanF1 were higher in the High than in the Low arousal condition, whereas SDF1, BWF1 and F2-F1 were lower in the High versus the Low arousal condition (t(17)≥|2.13|, N=18, p≤0.048). Furthermore, the consistency was lower in the High compared to the Low arousal condition (t(17)=3.03, N=18, p=0.008). For the tonality-related parameters two out of three parameters differed significantly between arousal conditions. Thus, the percentage of voiced frames and MaxHNR were lower in the High compared to the Low arousal condition (t(17)≥|2.51|, N=18, p=0.022; Figure 1).

**Table 3:** Mean and standard deviation of the acoustic parameters for Low and High arousal condition, results of the dependent t-test comparing both arousal-levels for each acoustic parameter and the correlation coefficient with the PC1; bold p-values represent significant difference; ↑ value is higher in the High than in the Low arousal condition, ↓ value is lower in the High than in the Low arousal condition; bold loading factors represent the parameters showing loading factors higher than 0.700 with the respective PC

	**LOW**		**HIGH**		**LOW versus HIGH**			
**Parameters**	**Mean**	**SD**	**Mean**	**SD**	**T**	**p**		**PC1**
**Time-related parameters**								
Call duration [ms]	566.34	168.62	707.10	186.09	−2.81	**.012**	↑	**-.756**
ICI [ms]	2072.53	1442.76	1075.38	652.32	2.58	**.019**	↓	.482
Peak time [ms]	0.23	0.08	0.29	0.12	−1.92	.072		-.640
**Source-related parameters**								
MeanF0 [Hz]	1305.42	238.49	1105.10	184.60	3.82	**.001**	↓	**.746**
MinF0 [Hz]	931.71	274.86	746.65	166.58	3.12	**.006**	↓	**.785**
MaxF0 [Hz]	1517.21	249.64	1316.52	221.48	3.68	**.002**	↓	.686
SDF0 [Hz]	154.99	37.51	149.56	37.23	.50	.623		-.077
**Filter-related parameters**								
Peak [Hz]	1648.68	327.77	2493.48	676.96	−5.24	**<.001**	↑	-.610
MeanF1 [Hz]	2112.80	420.47	2642.38	325.62	−4.84	**<.001**	↑	-.509
SDF1 [Hz]	696.02	273.16	549.43	159.78	2.13	**.048**	↓	.107
BWF1 [Hz]	1120.22	466.85	623.80	376.95	3.60	**.002**	↓	.442
MeanF2 [Hz]	7034.30	570.61	6758.39	511.89	1.79	.091		.095
SDF2 [Hz]	981.97	286.65	987.88	297.99	-.07	.948		-.247
BWF2 [Hz]	1977.26	512.96	1858.01	732.96	.75	.463		-.067
MeanF3 [Hz]	11320.63	552.86	11240.16	524.16	.47	.642		.118
SDF3 [Hz]	1134.20	249.39	1273.83	231.80	−1.52	.148		-.588
BWF3 [Hz]	2044.74	1128.99	3017.33	1602.68	−2.03	.058		-.410
F2-F1 [Hz]	4921.50	694.44	4116.01	745.54	4.31	**<.001**	↓	.348
Consistency	0.89	0.02	0.86	0.03	3.03	**.008**	↓	.312
**Tonality-related parameters**								
Cepstral peak [V]	2.36	0.59	2.69	0.61	−1.69	.110		-.366
Voiced [%]	98.23	1.71	96.26	2.67	2.53	**.022**	↓	**.712**
MaxHNR [db]	31.73	4.61	28.78	3.27	2.51	**.022**	↓	.576

**Figure 2:**
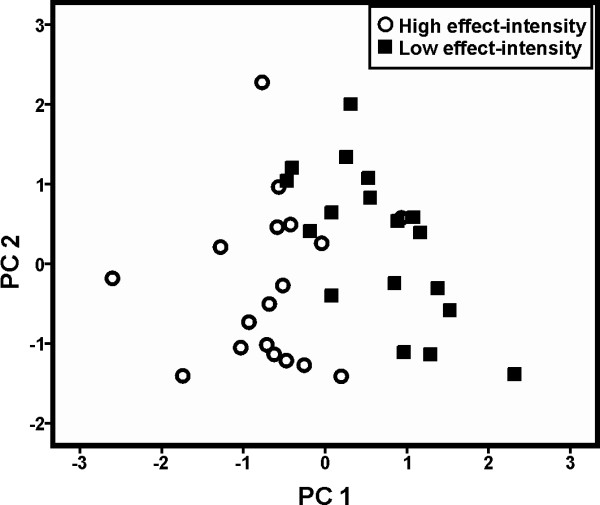
Scatterplot for the PC1 and PC2 of the arousal analysis.

Based on the means of the acoustic parameters for each individual and arousal condition a PCA extracted six factors with an eigenvalue higher than 1 explaining 81.28% of the variance (see Additional file 1). An independent DFA based on these six PCs was able to assign 88.9% of the cases to the respective arousal condition (cross-validation: 80.06%), which was significantly above chance level (50%; for original and cross-validated classification: both conditions: binomial test: p<0.001; Low arousal: p=0.008; High arousal: p=0.031; Figure 2). Thereby, PC1 showed the highest correlation with the discriminant function (r=0.709), whereas the other factors showed correlations lower than |0.219|. PC1 showed the highest loading factors to call duration (r=-0.756), MinF0 (r=0.785), MeanF0 (r=0.746) and percentage of voiced frames (r=0.712; Figure 1).

Analysing non-linear phenomena we detected NLPs in 47.46% of the analysed calls, but the percentage of calls containing NLPs was not significantly different between the Low and the High arousal condition (mean_Low_=50.00%; mean_High_=45.00%; Z=−0.358, n=17, N=18, p=0.720). The most often seen NLP was chaos (33.61%, N=18), followed by frequency jumps (15.43%, N=14) and subharmonics (9.26%, N=8). We found no significant differences in the percentage of calls containing frequency jumps (mean_Low_=20.00%; mean_High_=10.56%; Z=−1.84, n=12, N=18, p=0.066) or chaos (mean_Low_=38.89%; mean_High_=28.89%; Z=−1.03, n=15, N=18, p=0.304) between the Low and the High arousal condition. In contrast, subharmonics were only observed in the High and not in the Low arousal condition (mean_Low_=0.00%; mean_High_=18.33%; Z=−2.55, n=8, N=18, p=0.011).

Altogether, arousal conditions differed in time-, source-, filter- and tonality-related parameters. However, for classification the most loading acoustic parameters were call duration, percentage of voiced frames, mean and minimum fundamental frequency. In the High arousal condition significantly more calls containing subharmonics could be observed, whereas the occurrence of other NLPs did not differ between the two arousal conditions.

## Discussion

The results clearly show that in kitten isolation calls sender-identity and arousal-level are encoded by different combinations of acoustic parameters. Although univariate analysis showed that almost all kinds of acoustic parameters varied between sender-identity and arousal, DFA combined with PCA suggested that the impact of certain parameters differed. Sender-identity was mainly determined by a combination of source- and filter-related parameters, whereas arousal level was mainly determined by a combination of time-, source- and tonality-related parameters.

### Sender-identity

Kitten isolation calls differed between individuals in almost all acoustic parameters independent of arousal condition and could correctly be classified above chance level, supporting our hypothesis that sender-identity is encoded in the acoustic structure of kitten isolation calls. Analysis showed that this cannot be explained by the fact that we used a varying number of kittens per litter so that one litter can contribute more to the results than another. Thus, the pDFA controlling for litter also revealed differences in the acoustic structure between kittens.

Individual distinctiveness was found for both arousal conditions and could also be approved by pooling both conditions using a pDFA. Thereby, for both arousal conditions almost the same source- and filter-related parameters (MeanF0, MaxF0, F2-F1) contributed mainly to the classification result. This suggests that individual differences are consistent across different arousal levels. This is in agreement with several studies showing that infant isolation calls contain individual signatures (e.g., [16,62-64]). It can be assumed that these variations in the acoustic structure of kitten isolation calls can be perceived by the mother, since Härtle [55] demonstrated that mothers recognise their kittens from their voices. Thus, individual signatures in infant isolation calls would allow the mother to discriminate their own infant from those of others, to direct their care-giving behaviour and thereby increase their own fitness. This suggests that these individual signatures in kitten isolation calls may be an important tool for kin selection.

We found no effect of sex on the acoustic structure of kitten isolation calls, this being in agreement with other studies on small-bodied animals (e.g., tree shrews: [7]; pygmy marmosets: [65]; bats: [66]), whereas the majority of studies on large-bodied animals revealed sex-specific differences (see review on primates: [67]). Ey and colleagues [67] argued that these sex-specific differences were mainly driven by differences in body size due to sexual dimorphism. Since the kittens at this age did not show such a sex dimorphism in body weight, no differences in the acoustic structure of kitten isolation calls was expected. We also found no influence of body weight, which is also in agreement with findings of other studies (e.g., see review on primates [67] and additionally tree shrews: [7]). Ey and colleagues [67] argued that a relationship between body size and acoustic parameters is highly predictable when body size variation is large but less predictable if variation is small. Thus, it could be argued that the variation in body weight is not large enough to affect acoustic structures of vocalisations in kitten isolation calls (mean=307.33 g; range: 246–370 g; SD=33.03). All in all, kitten isolation calls contain individual signatures, which cannot be explained by sex or body weight.

### Arousal

Our hypothesis that arousal is encoded in acoustic parameters of kitten isolation calls was supported. Calls recorded in the High arousal condition were characterised by longer call duration, a shorter intercall-interval, a lower fundamental frequency, a higher peak- and first formant frequency and lower tonality values than calls recorded in the Low arousal condition. This is partly in agreement with other studies in cats investigating whether acoustic structure of isolation calls varies between contexts [58,60]. Our results are in line with the finding of Haskins [60] and Romand and Ehret [58] that call duration was shorter in low arousal contexts (Isolation without manipulation) than in high arousal context comparable to our High arousal condition (namely a Restrain context [60], Picked-up and Tail-pressing context [58]). Regarding our finding that the fundamental frequency was decreased in the High arousal condition in comparison to the Low arousal condition, our data are not in agreement with those of Haskins [60] who found no significant differences in the fundamental frequency between the Isolation and the Restrain context. However, Romand and Ehret [58] found that the fundamental frequency became significantly lower in the Tail-pressing context than in the Isolation context when kittens turned 32 days old.

Comparing our results with other animal taxa we found that for the temporal parameters similar changes are reported for a variety of mammalian taxa and behavioural contexts (see review [23,24]). Concerning source-related parameters the results are controversial. Thus, the majority of studies found either an increase of fundamental frequency with increasing arousal or no effect (see review [23,24]). Surprisingly, we found a decrease in fundamental frequency from Low to High arousal condition. As described above, also Romand and Ehret [58] found a decrease in F0 from the Tail-pressing context (similar to our High arousal condition) compared to the Isolation context (similar to our Low arousal condition) in 32–46 day-old kittens. Furthermore, during male-male interaction it was shown for grey mouse lemurs that the start fundamental frequency of their calls was lower in contexts where they had physical fights (assumed to reflect high arousal) compared to contexts where they had no physical contact (assumed to reflect low arousal in the animal) [68].

For the filter-related parameters we found an increase of the peak frequency and the frequency of the first formant from Low to High arousal condition. An increase in the frequency of filter-related parameters was also found for pigs [69], primates [30,70] and tree shrews [7]. An increase in the frequency of the first formant (=resonance frequency) was also found in pigs [71] and chimpanzees [70]. Furthermore, a decrease in the consistency agrees with findings in tree shrews [7]. The increase in peak frequency and formant frequencies could be explained by the extent of mouth opening which results in a shorter vocal tract length [72]. It could be argued that the changes we found for the acoustic parameters, especially those of filter-related parameters, could be attributed to the manipulation in the High arousal condition. This means by turning the kittens on their back the length of the vocal tract may be changed. However, we did not systematically manipulate the head position so that the angle between the head and the breast could vary between kittens. Due to this unsystematical variation of head position, it would be unlikely that the analysis of sender-identity favoured the same source- and filter-related parameters for both arousal conditions. Thus, we suggest that turning the kitten onto its back cannot account for the increase in filter-related parameters. Instead, we favour the assumption that mouth opening shortens the vocal tract, resulting in an increase of filter-related parameters which was already shown for cats by Shipley and colleagues [72]. The decrease in tonality from Low arousal to High arousal condition agrees with findings in other animals (e.g., [7,20,37]). The decrease in tonality may go along with an increase in non-linear phenomena due to a loss of vocal control [37]. However, we found only a difference in the percentage of calls containing subharmonics between the Low- and the High arousal condition but not for NLPs in general, chaos or frequency jumps. Stoeger and colleagues [37] found a positive correlation between harmonic-to–noise ratio (HNR) and duration of chaotic segments. Since we found a decrease in the MaxHNR it could be assumed that although the occurrence (percentage of calls) is the same the relation of NLP in the call differs. In the data set we used for these analyses we could not always decide reliably when a chaotic component started or finished. Therefore, further studies are needed to investigate the role and function of non-linear phenomena in kitten isolation calls.

To expose animals to a situation assumed to induce a specific emotion and measuring the corresponding behavioural and physiological changes is a general approach in animal emotional research [24]. Vocal correlates of arousal were investigated by exposing subjects to different levels of situational urgency within the same behavioural context and analysing the acoustic parameters of their vocal expressions (e.g., [7,23,30,34,44]). In this study kittens were separated from their mother and siblings in both conditions. In the Low arousal condition they were left undisturbed whereas in the High arousal condition they were additionally manipulated by the experimenter assumed to induce a higher level of urgency/arousal. However, although if we assume that the general behavioural context and the emotional quality might be fairly similar between the sub-contexts, we can not rule out that the meaning/function of vocalizations differs between sub-contexts. To clarify this, further studies are needed which expose kittens to different contexts assumed to vary in arousal and also in emotional quality and compare their responses.

All in all, we found that arousal-related changes of time- and tonality-related parameters in kitten isolation calls correspond with previous findings in other mammalian taxa.

## Conclusion

In conclusion, our results showed that kitten isolation calls encode sender-identity and arousal. Thereby, different sets of parameters seem to be important. Thus, time-, source- and filter-related parameters mainly encode for arousal, whereas source- and filter-related parameters mainly encode for sender-identity. Thereby, source-related parameters seem to be important for both coding the sender-identity and arousal. This suggests that based on parameters of the fundamental frequency alone we cannot differentiate between sender-identity and arousal. Instead, we argue that single parameters alone do not code for arousal and sender-identity (especially because all vary) but that certain sets or relations of parameters encode sender-identity or arousal. Thus, playback studies are needed, manipulating specific acoustic parameters, to verify which acoustic parameters are biologically important for recognising sender-identity and arousal.

## Material & methods

### Subjects and housing

We tested 18 mongrel kittens (9 males, 9 females) from 6 litters aged 9 to 11 days and housed in the SPF (Specific Pathogen Free) breeding colony at the Hannover Medical School. All kittens were reared by their mothers. The animal husbandry there complies with the recommendations for domestic cats noted in Appendix A of the European Convention for the Protection of Vertebrate Animals used for Experimental and other Scientific Purposes (ETS No.123) ( http://conventions.coe.int/Treaty/EN/Treaties/PDF/123-Arev.pdf). One mother and her kittens lived in one animal room (12.5 m^2 ^to 20.6 m^2^) equipped with a wooden nest box, an infrared lamp as additional heat source, bars for scratching and plastic items for playing. Cats were used to the daily routine of animal keepers entering the animal rooms and playing with or grooming them. All kittens were familiar with being handled by humans due to the daily weighing routine and mothers were used to the kittens being removed for a short time from the nest box. Furthermore, they had acoustic and olfactory contact to other cats. The mother was fed daily with canned (Pet, De Haan Petfood, Nieuwkoop, the Netherlands) and dry cat food (SDS Pet Food, Special Diets Services, Witham, Essex, UK). Additionally, freshly killed rats were provided daily together with milk or curd cheese. Water was available ad libitum. Animals were housed at a temperature of 22±2°C, relative humidity of 55±5% and a light/dark cycle of 12:12 hours (lights on at 6:00 a.m.).

### Experimental procedure and data recording

Experiments were performed in the animal rooms of the respective mother and her kittens. We conducted a separation paradigm in which each kitten was removed from its nestbox and spatially separated from its mother and siblings. To induce two different levels of arousal in a kitten (the Low and High arousal condition), kittens were exposed to two sub-contexts varying in the level of situational urgency. Thus, in the Low arousal condition a kitten was only spatially separated from its mother and siblings and left undisturbed by the experimenter (=placed alone on the floor of the animal room), whereas in the High arousal condition a kitten was additionally manipulated by the experimenter i.e. the kitten was grasped, lifted off the ground and/or turned onto its back so that the legs had no contact to the ground. In the Low arousal condition kittens moved around slowly, whereas in the High arousal condition they struggled with their legs and tried to turn around. Thus, we assume that the strong manipulation by the experimenter in the High arousal condition induced a higher level of urgency/arousal in the kitten compared to the Low arousal condition where they were left undisturbed.

Kittens were tested in one session. In this session both conditions were performed in a randomised order for 3 minutes each. After finishing a condition kittens were reunited with their mother and siblings before the other condition was performed. The inter-condition interval was dependent from the number of siblings. Thus, we tested the kittens of one litter one after another in the first condition. After finishing this test for all kittens we started to test the kittens in the same order for the second condition. To avoid stress for the mother, the mother remained in the animal room but was prevented from coming into contact with the kittens during the experimental trial by the animal keeper (e.g., groomed or played with the mother).

Kitten vocal responses were recorded using a Sennheiser microphone (ME 67, Sennheiser, Wedemark, Germany; frequency range: 40 – 20,000 Hz) linked to a Marantz professional solid state recorder (PMD 660, Marantz, Osnabrück, Germany; sampling frequency: 44.1 kHz, 16 bit). Sound files were stored as wave files on a Compact Flash memory card (4 GB, Scan Disk Corporation, Milpitas, CA, USA). The kittens’ behaviour were videotaped using a digital camcorder (Sony DR-TRV 22E-PAL, Tokyo, Japan).

### Acoustic analysis

**Figure 3:**
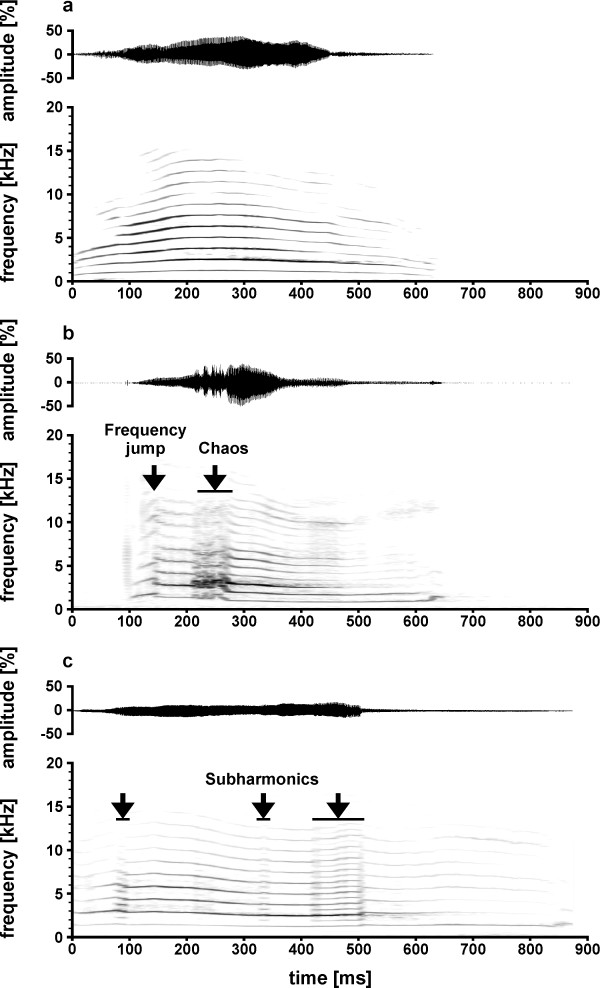
Example of kitten isolation calls; (a) harmonic isolation call without non-linear phenomena, (b) isolation call with a frequency jump and a chaotic component, (c) isolation call with subharmonics.

Vocal recordings were visually inspected using spectrograms of the software Batsound PRO 3.31 (Pettersson Elektronik AB, Uppsala, Sweden). Isolation calls were characterised as tonal calls with a rise and fall in the fundamental frequency with peak intensity around the mid-point (Figure 3a; [57]). For each individual and each arousal condition we selected 10 calls of good quality with a minimum amplitude difference of 5% between background noise and maximum amplitude of the call. For the Low arousal condition we selected the first 10 calls of good quality. For the High arousal condition we selected the first 10 calls of good quality after turning the kitten onto its back (except for one kitten which was only lifted up so that its legs had no contact to the ground). In total, we analysed 348 calls from 18 individuals. For two individuals only three and five calls were available in the Low arousal condition.

We performed a multi-parametric sound analysis using the software Batsound PRO 3.31, SIGNAL 3.1 (Engineering Design, Berkeley, California, U.S.A.) and PRAAT ( http://www.praat.org; [73]) combined with GSU PRAAT TOOLS [74]. The software Batsound PRO was used to manually measure the call duration and intercall-interval using the oscillogram of the calls. Furthermore, we classified visually whether a call contained NLPs and which type of NLP was present. According to the classification of Riede and colleagues [35] we classified calls as calls containing NLPs if we could observe one or more of the following non-linear components: Frequency jumps, subharmonics or chaos (Figure 3b-c). Frequency jumps were defined as abrupt upward and downward transitions of the fundamental frequency (F0). Subharmonics were defined as additional spectral components at integer fractions of the F0 (e.g., F0/2, F0/3). Chaos was defined as broad-banded frequency components which could contain traces of harmonic elements. If a call contained none of these components we classified it as a harmonic call (Figure 3a). To control for reliability of visual classification, a second person analysed all calls and we calculated the percentage of agreement between both persons. For NLPs in total both persons agreed in 85.63% of the calls, for frequency jumps in 87.64% of them, for chaos 85.63% of them and for subharmonics in 95.98% of them, respectively. The software SIGNAL 3.1 was used to measure the peak frequency, the cepstral peak and consistency using self-written macros. We calculated a power spectrum over the entire call to measure the peak frequency. To measure the cepstral peak, we calculated the cepstrum over the 10 ms in the middle of the call. The cepstrum is a spectrum of the signal (=cepstrum, CEP command), which is used to study the periodicity of a time signal. The cepstrum shows a cepstral peak at periodicity of a signal (=harmonic interval of the signal). Thus, a signal with a fundamental frequency of 100 Hz shows a cepstral peak at 10 ms (1/ 100 Hz=10 ms). The cepstral peak is higher for calls with a clear harmonic structure (high tonality) and a stable pitch [20]. To measure spectral consistency across the entire call we measured the maximum correlation by correlating power spectra of successive 10 ms time segment of the entire call with each other. The maximum correlation is the maximum value of the normalised cross-covariance function which is a sequence of correlation values for successive intervals. The software PRAAT combined with GSU PRAAT TOOLS 1.9 (GSU -> quantify) were used to measure acoustic parameters related to fundamental frequency, formants and tonality-related parameters. Using the sub-menu “quantify Amp and Dur”, the Peaktime, i.e. time between the onset and the maximum amplitude of the call, was measured. Using the sub-menu “quantify Source” (min pitch: 75 Hz; max pitch: 3000 Hz; time steps: 0.01 s) the source-related parameters as well as the number of voiced frames (Voiced) and the maximum harmonic-to-noise-ratio (MaxHNR) were measured. We used the pitch target segment to check and correct the data. Using the sub-menu “quantify formant” (number of formant: 4; max formant value: 20 kHz; time steps: 0.01 s; see Additional file 2) we measured the first, second and third formant. To estimate the number of formants expected in kitten isolation calls we used a formula according to Pfefferle and Fischer [75].

(1) $$N=\left(\frac{2\times L}{C}\times f_{c}\right)$$

where N=number of formants, L=vocal tract length [m], c=speed of sound (350 m/s) and f_c_=cut-off frequency [Hz]. Carterette and colleagues [76] reported the length of the vocal tract for young kittens (first week of life) as being approximately 3.0 to 3.5 cm. As we tested kittens of 9–11 days, we used the maximum value of vocal tract length, reported by Carterette and colleagues [76], for estimating the number of formants (L=3.5 cm). Kitten isolation calls ranged up to a frequency of 20 000 Hz, which we used as the cut-off frequency. Furthermore, we calculated the distance between the mean of the second and the first formant.

In total, we measured 3 time-, 4 source-, 12 filter- and 3 tonality-related parameters. Detailed descriptions of the acoustic parameters are presented in Table 1.

### Statistical analysis

To analyse whether the order in which subjects were exposed to the two conditions effects the acoustic parameters of their vocalisations we performed independent t-tests and controled for multiple testing by applying the Fishers Omnibus test combining multiple p-values [77].

To investigate sender-identity in kitten isolation calls, we conducted the following analysis for each condition separately: First, to investigate whether acoustic parameters differ statistically between individuals we performed a One-way-ANOVA. To control for multiple testing we applied the Fishers Omnibus test combining multiple p-values [77]. Second, to investigate whether calls can correctly be classified to the respective individuals, we performed an independent DFA combined with a PCA. Thus, we first performed a PCA extracting PCs with an eigenvalue higher than 1 to reduce the number of parameters. We considered acoustic parameters with a loading factor higher than 0.700 to the respective PC as parameters, which have a strong impact on this factor. Based on these extracted PCs we calculated a DFA. In addition to the DFA original classification, we performed a cross-validation using the leave-one-out method. Furthermore, we investigated whether the number of correctly classified cases was significantly higher than expected by chance using a binomial test.

To investigate whether the level of individual distinctiveness may vary between arousal conditions, we recalculated the DFA for the High arousal condition using the same subjects as for the Low arousal condition (N=16) and compared the percentage of correctly classified calls per subject between arousal conditions using the dependent t-test. To test the consistency of individual signatures across arousal levels, we pooled the data for both arousal conditions and performed a crossed permutated DFA (pDFA; [78]) using subject as test factor and arousal as control factor. Since subjects belong to different litters and litter size differs, we also performed a nested pDFA using subject as test factor and litter as control factor.

To control for the effect of sex and body weight on the acoustic structure of kitten isolation calls, we conducted independent t-tests comparing the acoustic parameters between male and female kittens for each acoustic parameter as well as body weight and correlated body weight with the acoustic parameters using a Pearson correlation.

To investigate whether arousal is encoded in kitten isolation calls we first calculated the mean of each acoustic parameter and condition for each individual. Then we compared each of these means between the Low and High arousal condition using a dependent t-test. To test whether arousal could be correctly classified based on the acoustic parameters of the isolation calls we conducted an independent DFA based on the means of the acoustic parameters for each subject similar to the sender-identity analyses (see above).

To investigate the occurrence of non-linear phenomena, for each individual we calculated the percentage of calls containing NLPs (total), frequency jumps, chaos, or subharmonics. To investigate whether the occurrence of NLPs differed between conditions, we compared these percentages between conditions using a non-parametric test, the Wilcoxon Signed Rank test, because these data were not normally distributed.

All tests were performed using the statistical software SPSS 19 except the Fisher Omnibus test and the pDFA. The Fisher Omnibus test was calculated manually using Excel. The pDFA was performed using scripts written by R. Mundry (MPI for Evolutionary Anthropology, Leipzig, Germany) which runs in the statistical software R ( http://www.r-project.org/).

## Abbreviations

NLP: Non-linear phenomena; F0: Fundamental frequency; PCA: Principal component analysis; PC: Principal component factor; DFA: Discriminant function analysis; DF: Discriminant function; ICI: Intercall-interval; MeanF0: Mean fundamental frequency; MinF0: Minimum fundamental frequency; MaxF0: Maximum fundamental frequency; SDF0: Standard deviation of fundamental frequency; Peak: Peak frequency; MeanF1: Mean frequency of the first formant; SDF1: Standard deviation of the first formant; BWF1: Bandwidth of the first formant; MeanF2: Mean frequency of the second formant; SDF2: Standard deviation of the second formant; BWF2: Bandwidth of the second formant; MeanF3: Mean frequency of the third formant; SDF3: Standard deviation of the third formant; BWF3: Bandwidth of the third formant; F2-F1: Difference between second and first formant frequencies; Voiced: Percentage of voiced frames; MaxHNR: Maximum harmonic-to-noise ratio.

## Authors’ contributions

MS designed the study, conducted the experiments, performed the acoustic and statistical analyses and prepared the manuscript. AR participated in data collection and conducted preliminary acoustic and statistical analyses. WK participated in data collection and contributed to the preparation of the manuscript. EB, HH and EZ initiated the study and contributed to the preparation of the manuscript. All authors read and approved the final manuscript.

## Supplementary Material

Additional file 1 **Summary table of the results of the PCA and DFA for sender-identity and arousal.** Results for the analysis of sender-identity are separated for both arousal conditions LOW=Low arousal condition; HIGH= High arousal condition; No. of PCs = number of PCs with an eigenvalue higher than 1; Explained variance = percentage of variance which can be explained by the PCs with an eigenvalue higher than 1; No. of DFs = number of DFs which were calculated; For the original classification and for the cross-validation the percentage of correctly classified calls (=Correctly classified calls), the significance value of the binomial test testing whether the number of correctly classified calls was above chance (=Binomial test) are provided. Correlation between DFs and PCs = PC which showed highest correlation with the respective DF (loadings for the acoustic parameters with the respective PC are shown in Tables 2 and 3). (DOC 35 kb) Click here for file

Additional file 2 **Spectrogram of a kitten isolation call indicating formants.** Formants marked by red dots; Spectrogram setting: window length=0.001; Formant settings: Maximum formant=20.000 Hz, Number of formants=4, window length=0.025. Click here for file
