# Supplementary material for: Vocal correlates of sender-identity and arousal in the isolation calls of domestic kitten (Felis silvestris catus)
Source: Front Zool. 2012 Dec 21;9:36. doi: 10.1186/1742-9994-9-36 (PMC3551667; doi:10.1186/1742-9994-9-36)
Supplement: Additional file 1 — Summary table of the results of the PCA and DFA for sender-identity and arousal. Results for the analysis of sender-identity are separated for both arousal conditions LOW=Low arousal condition; HIGH= High arousal condition; No. of PCs = number of PCs with an eigenvalue higher than 1; Explained variance = percentage of variance which can be explained by the PCs with an eigenvalue higher than 1; No. of DFs = number of DFs which were calculated; For the original classification and for the cross-validation the percentage of correctly classified calls (=Correctly classified calls), the significance value of the binomial test testing whether the number of correctly classified calls was above chance (=Binomial test) are provided. Correlation between DFs and PCs = PC which showed highest correlation with the respective DF (loadings for the acoustic parameters with the respective PC are shown in Tables 2 and 3). (DOC 35 kb) [file 1742-9994-9-36-S1.doc]

Additional File 1:

**Summary table of the results of the PCA and DFA for sender-identity and arousal**

Results for the analysis of sender-identity are separated for both arousal conditions LOW=Low arousal condition; HIGH= High arousal condition; No. of PCs = number of PCs with an eigenvalue higher than 1; Explained variance = percentage of variance which can be explained by the PCs with an eigenvalue higher than 1; No. of DFs = number of DFs which were calculated; Chance level = a-priori probability for classification; For the original classification and for the cross-validation the percentage of correctly classified calls (=Correctly classified calls), the significance value of the binomial test testing whether the number of correctly classified calls was above chance (=binomial test) as well as the number of individuals for which the classification was significantly above chance level (=individual level) are provided. Correlation between DFs and PCs = PC which showed highest correlation with the respective DF (loadings for the acoustic parameters with the respective PC are shown in Tables 2 and 3).

|  | Sender-identity | | Arousal |
| --- | --- | --- | --- |
|  | LOW | HIGH |  |
| No. of subjects | 16 | 18 | 18 |
| PCA | | | |
| No. of PCs | 7 | 7 | 6 |
| Explained variance | 71.95 % | 68.9 % | 81.28 % |
| DFA | | | |
| No. of DFs | 7 | 7 | 1 |
| Chance level | 6% | 6% | 50% |
| **Original classification** |  |  |  |
| Correctly classified calls | 53.13 % | 63.33 % | 88.9 % |
| Binominal test | <0.001 | <0.001 | <0.001 |
| **Cross-validation** |  |  |  |
| Correctly classified calls | 41.88 % | 47.78 % | 80.06 % |
| Binominal test | <0.001 | <0.001 | <0.001 |
| Correlation between DFs and PCs | DF1 ~ PC1  DF2 ~ PC6  DF3 ~ PC2 | DF1 ~ PC1  DF2 ~ PC2  DF3 ~ PC3 | DF1 ~ PC1 |
