# Supplementary figures and images for: Vocal correlates of sender-identity and arousal in the isolation calls of domestic kitten (Felis silvestris catus)
Source: Front Zool. 2012 Dec 21;9:36. doi: 10.1186/1742-9994-9-36 (PMC3551667; doi:10.1186/1742-9994-9-36)

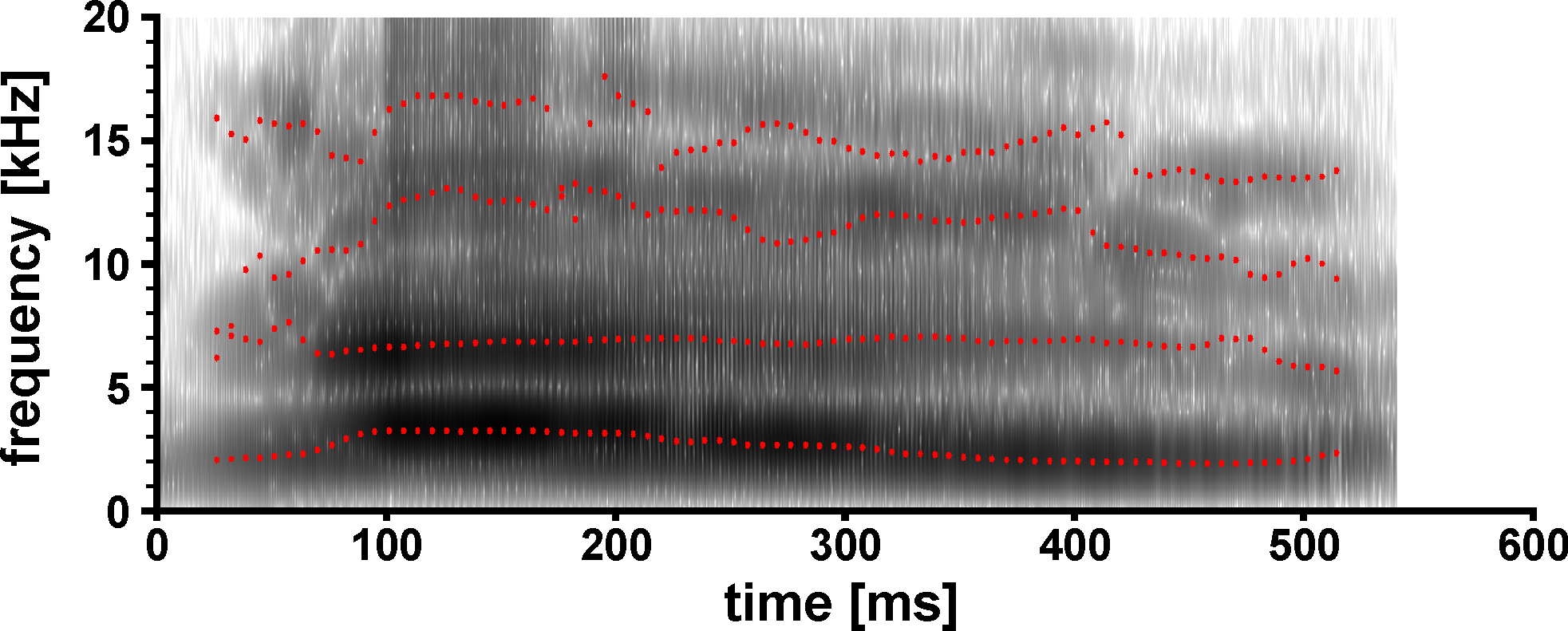

Supplement: Additional file 2 — Spectrogram of a kitten isolation call indicating formants. Formants marked by red dots; Spectrogram setting: window length=0.001; Formant settings: Maximum formant=20.000 Hz, Number of formants=4, window length=0.025. [file 1742-9994-9-36-S2.tiff]
